# Secret Forwarding of Events over Distributed Publish/Subscribe Overlay Network

**DOI:** 10.1371/journal.pone.0158516

**Published:** 2016-07-01

**Authors:** Young Yoon, Beom Heyn Kim

**Affiliations:** 1 Department of Computer Engineering, Hongik University, Seoul, South Korea; 2 Department of Computer Science, University of Toronto, Toronto, Canada; Tianjin University of Technology, CHINA

## Abstract

Publish/subscribe is a communication paradigm where loosely-coupled clients communicate in an asynchronous fashion. Publish/subscribe supports the flexible development of large-scale, event-driven and ubiquitous systems. Publish/subscribe is prevalent in a number of application domains such as social networking, distributed business processes and real-time mission-critical systems. Many publish/subscribe applications are sensitive to message loss and violation of privacy. To overcome such issues, we propose a novel method of using secret sharing and replication techniques. This is to reliably and confidentially deliver decryption keys along with encrypted publications even under the presence of several Byzantine brokers across publish/subscribe overlay networks. We also propose a framework for dynamically and strategically allocating broker replicas based on flexibly definable criteria for reliability and performance. Moreover, a thorough evaluation is done through a case study on social networks using the real trace of interactions among Facebook users.

## Introduction

Publish/Subscribe (in short pub/sub) is a communication paradigm where loosely-coupled clients communicate in an asynchronous fashion. Subscribers issue subscriptions to express their interest in certain topics and/or content. Publishers disseminate their publications to the subscribers through a pub/sub routing system without directly being aware of their identities and/or locations [1]. Because of such asynchronous nature, pub/sub paradigm supports the flexible development of large-scale, event-driven and ubiquitous systems. Pub/sub is prevalent in many application domains such as distributed business activity monitoring [2], stock price monitoring for algorithmic trading systems [3], complex-event processing [4] and mission-critical systems such as air traffic control system [5]. A multi-national research group has adopted pub/sub routing paradigm to improve the architecture of Internet that recently exhibits more content-oriented communication patterns [6, 7]. Many social networking services are built around the pub/sub abstraction [8, 9]. Recently, notable international consortiums such as Allseen and OIC acknowledge pub/sub as the critical communication substrate for Internet of Everything (IoE) platforms, and naturally protocol standards such as MQTT and CoAP are receiving great attention from the IoE application developers who need to implement pub/sub communication [10, 11]. We can also envision potential applications of pub/sub systems in the study of complex networks that analyzes the patterns of connections between elements of real systems [12–16]. For instance, multivariate signals are measured from the distributed conductance sensors in order to analyze oil-water flow patterns, which are subsequently visualized in terms of community structure [17]. These sensors can be deployed on a pub/sub system so that interested patterns can be filtered and delivered in a more scalable and efficient way.

**Fig 1:**
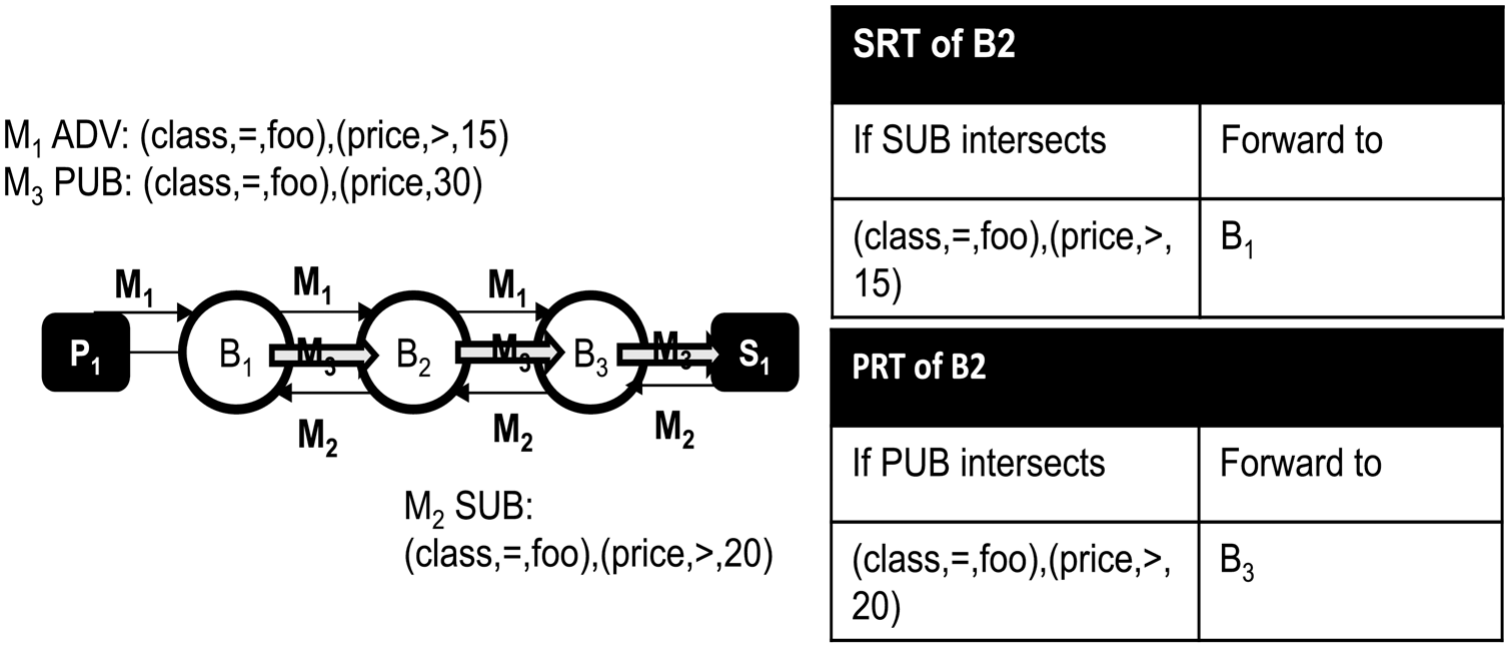
An example of routing state updates on pub/sub overlay.

Pub/sub systems are typically formed into an overlay of distributed event matching and forwarding brokers [8–11, 18, 19] in order to process a large volume of events in a scalable manner. In reference implementations of pub/sub broker overlay [20, 21], a publisher first disseminates an *advertisement* to all the brokers before publishing events. We call a published event as a *publication*. Publication can be labeled with a specific topic and can contain messages or content. If a subscription matches an advertisement in the SRT (Subscription Routing Table), which is essentially a list of [advertisement,last hop] tuples, the subscription is forwarded to the last hop broker where the advertisement came from. In this way, subscriptions are routed towards the publisher. Subscriptions are used to construct the PRT (Publication Routing Table). The PRT is a list of [subscription,last hop] tuples, which is used to route publications. If a publication matches a subscription in the PRT, it is forwarded to the last hop broker where the subscription came from. This process continues until the publication finally reaches the subscriber. Fig 1 shows an example of content-based routing. In Step *1*, an advertisement (*M*_1_) arrives at *B*_1_. In Step *2*, a matching subscription (*M*_2_) arrives at *B*_3_. Since *M*_2_ matches *M*_1_ at broker *B*_3_, *M*_2_ is relayed to *B*_1_ which is the last hop of *M*_1_. After the completion of these steps, PRTs are updated accordingly along the path ($\vec{p}$) from *B*_1_ to *B*_3_. Based on the routing information on the PRTs on $\vec{p}$, a publication (e.g., *M*_3_) that matches the subscription *M*_2_ can be delivered to the subscriber *S*_1_ through $\vec{p}$. Subscribers can specify an interest on a particular topic such as (class,=,bar) in *M*_2_. Subscribers can also express the interest in a more fine-grained way by being specific on the content. For example, *S*_1_ expressed the interest over particular value range for the attribute price, as shown in Fig 1.

Our major concern is that the pub/sub brokers can fail or be compromised and thus behave arbitrarily to hamper reliable and secure event delivery. We refer to an arbitrarily-behaving pub/sub broker as a *Byzantine* broker. Byzantine brokers can be present across and along many end-to-end delivery paths between publishers and subscribers. Any arbitrary behavior of the Byzantine brokers can subvert applications running on pub/sub overlays and lead to very harmful result to end-users. Therefore, we need to devise a novel solution that can effectively deal with this issue. Specifically, we aim to ensure the satisfaction of the following requirements, even under the presence of Byzantine brokers [22]. First, we must make sure that publication messages are delivered to the interested subscribers without any loss (a *reliability* requirement). Second, we should not let a subscriber or a Byzantine broker access the sensitive content in a publication message without access privilege (a *confidentiality* requirement).

As discussed further in the related work section, existing works for countering the violation of the aforementioned requirements typically employ replication and encryption techniques. Replicated brokers can decrease the possibility of message loss. Encryption can protect the private portion of publication messages. However, to the best of our knowledge, these works overlook the possibility of the decryption keys getting compromised and abused by Byzantine brokers. Byzantine brokers can drop the keys to prevent the interested subscribers from decrypting publication messages. Using the compromised keys Byzantine brokers may decrypt private publication messages and disclose them to unauthorized subscribers. Byzantine brokers can simply drop both the encrypted messages and the decryption keys. We need a solution that addresses such threats to the reliable and secure operation of pub/sub middleware.

In this paper, we present a novel method that applies the secret sharing technique [23] to a group of replicated brokers chained on a pub/sub overlay [24]. To give a high-level overview of our solution, broker replicas are first placed along the end-to-end paths between publishers and subscribers. Publishers split the decryption key by using the secret sharing scheme. Spit keys that we call *secret shares* are propagated along with the encrypted publication messages. The secret shares are generated and forwarded to the replicated brokers in such a way that the original decryption keys cannot be reconstructed by Byzantine brokers. With this method, confidential publication message is safe from being leaked. The replicas are used to prevent the Byzantine brokers from dropping publications and keys, or sending publications to unauthorized subscribers.

Our method may introduce increased performance overhead due to the addition of more brokers to the pub/sub overlay. Therefore, we also face the challenge of utilizing the given broker replicas in the most efficient manner. To address these challenges, we propose a framework for dynamically and strategically allocating broker replicas based on reliability and performance criteria that can be defined flexibly by pub/sub overlay administrators.

We evaluated the effectiveness of our solution by applying it to a service that publishes the interactions that happened in a social network service. Specifically, we retrieved the real trace of interactions among anonymous Facebook users and re-played the trace on the pub/sub middleware equipped with our new solution.

The rest of the paper is organized as follows. First, we present the details of our secret forwarding method and discuss various adaptations. Second, we describe the framework for allocating replicas according to dynamically changing demand on reliability and performance. Third, we analyze the performance evaluation result. Finally, we discuss related works and conclude.

## The Secret Forwarding Method

In this section, we introduce a method that guarantees a reliable and confidential delivery even under the presence of Byzantine brokers. The main point of our solution is to enforce a secret-sharing scheme [23] for securely delivering decryption keys.

### Secret Sharing in a Pub/Sub Overlay

One of our major concerns is that Byzantine brokers may arbitrarily process the encrypted publication messages using a compromised decryption key. Therefore, it is imperative that the key should be protected from the Byzantine brokers. Here, we employ Shamir’s secret sharing scheme [23]. With this technique, a secret can be split into *n* shares in such a way that at least *k* shares are needed to reconstruct the original secret. In other words, even if up to *k*-1 shares are compromised, the original secret cannot be re-generated. This is called the (*k*, *n*) threshold scheme with the constraint that *k* > 1 and *n* ≥ *k*. In our context, the secret is the key necessary for subscribers to decrypt the publication messages that are encrypted by publishers. In this section, we focus on the case where the Byzantine brokers reside along the end-to-end path between publishers and subscribers. We show how the original decryption key should be split by a publisher assuming that there is up to *f* number of Byzantine brokers at the next immediate hop. Then, we explain how the split secret shares should be propagated towards the interested subscribers.

#### Initial Key Split

**Fig 2:**
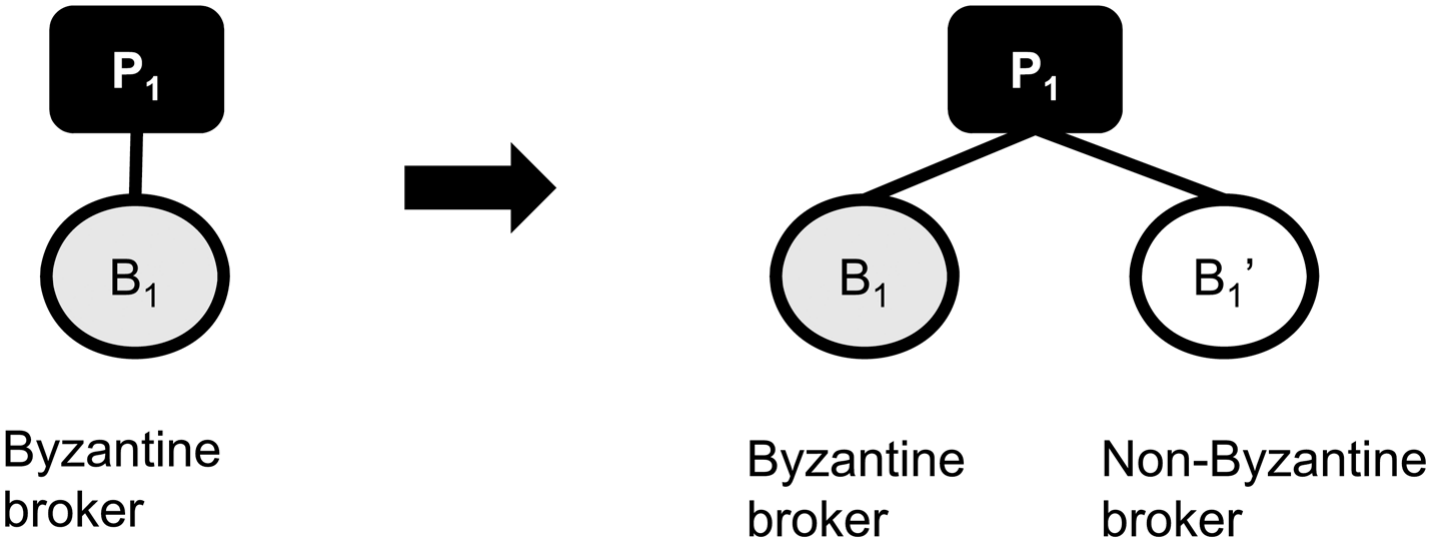
A simple broker replication example for handling the case where a Byzantine broker violates the reliable publication delivery requirement.

Suppose the publisher *P*_1_ sends out a publication *p*_1_ via broker *B*_1_ as shown in Fig 2. Assume that *B*_1_ is the Byzantine broker and drops *p*_1_. In order to prevent the loss of messages, a redundant path can be established via replica $B_{1}^{\prime }$. Duplicate publications can be sent through the redundant paths so that at least one message is guaranteed to be forwarded towards the subscribers. With the replica, the pub/sub overlay becomes tolerant to a single failure of reliable delivery. As a generalization, if there are *f* failures at each hop on an overlay, at least *f* + 1 replicas are needed on that hop. These replicas form a group that we call a *virtual node*.

For decryption keys, we use the secure *in-band* key delivery strategy. Similar to the previous case, we need to add broker replicas in order to tolerate node failures. However, the simple replication technique we used in the previous example is not sufficiently safe for the case of transferring the decryption key. The difficulty stems from the fact that it is impossible to perfectly detect whether a broker is Byzantine or not. In order to prevent the Byzantine brokers from obtaining the decryption keys, we choose to employ the secret sharing technique to safely split the decryption keys into multiple shares, initially at the publishers. Assume that there are *r* replicas in the virtual node *V* which is the next hop of publisher *P*. The brokers in *V* to which the publishers are directly connected are referred to as *publisher-edge brokers*. A secret can be split into *r* shares by a publisher, and these shares can be evenly distributed among the *r* replicas at *V*. Having only one secret share, each replica cannot reconstruct the original secret. However, we cannot rule out the possibility of multiple Byzantine brokers colluding to collect a sufficient number of shares required for the reconstruction of the original secret. Also, assuming that (*k*, *n*) threshold scheme is used by the publishers, the *f* Byzantine brokers among the publisher-edge brokers may drop *k* secret shares. Even if other non-Byzantine brokers correctly deliver *k*-1 secret shares to the authorized subscribers, those secret shares are not sufficient for reconstructing the secret decryption key. Based on this observation, we have to first assume that the number of Byzantine brokers should be less than *k* in order to prevent these brokers from breaking the (*k*, *n*) threshold scheme and the requirement of reliably delivering the secret decryption key to the authorized subscribers.

This assumption is more formally stated in Assumption 1 as follows.

**Assumption 1** *At every virtual node V, there are* $\lfloor \frac{\left|V\right|}{2}\rfloor +1$ *brokers that are non-Byzantine*.

For example, if there are 5 brokers in a virtual node, we assume that there are up to 2 ($=\lfloor \frac{5}{2}\rfloor$) Byzantine brokers and there are at least 3 ($=\lfloor \frac{5}{2}\rfloor +1$) non-Byzantine brokers.

Assumption 1 reflects the maximum fault-tolerance we aim to achieve. This is a reasonable assumption given the following threat model. Each server running a broker replica follows independent authentication and authorization process, thus a security breach on a particular server does not immediately and/or automatically lead to another security breach on other servers.

Given Assumption 1 and the (*k*, *n*) threshold scheme, we have to ensure that *k* secret shares among the *n* secret shares must be delivered only to *k* non-Byzantine brokers. In other words, if there are *f* Byzantine brokers, there have to be at least *f* + 1 additional replicas that are non-Byzantine brokers. Therefore, the threshold-scheme to be used at the publisher can be expressed as (*f* + 1, 2*f* + 1). Alternatively, we can express the threshold scheme as ($\lfloor \frac{n}{2}\rfloor +1$, *n*) where *n* is the number of replicas in the next-hop virtual node. For example, if there are 5 publisher-edge brokers at the next hop of a publisher *P*, then a (3, 5) threshold scheme should be used by *P*.

However, the initial key split alone does not guarantee that the secrete shares can be safely delivered to the subscribers beyond the publisher-edge brokers. We articulate this problem further in the following subsection.

#### Propagation of Secret Shares

Before we present the problem of reliably propagating the secret shares to the subscribers, we enlist key notations as follows.

- *V*: A virtual node
- *V*_*f*_: A virtual node with forwarding brokers
- *V*_*r*_: A virtual node with receiving brokers
- *prec*(*V*): A virtual node that precedes *V*, e.g., *V*_*f*_ precedes *V*_*r*_
- |*V*|: The number of brokers in virtual node *V*
- *reconstruct*(S): A predicate that returns true if a currently received set of split secret shares S can be used to reconstruct the secret split at the preceding virtual node *V*_*p*_.
- *nByz*: A non-Byzantine broker
- *Byz*: A Byzantine broker
- *nByz*(*V*): A set of non-Byzantine brokers in *V*
- *Byz*(*V*): A set of Byzantine brokers in *V*
- *B*(*V*): A set of all brokers in *V*

The first challenge is to prevent Byzantine brokers from tampering with the secret shares it received from the previous hop. Such tampering can be trivially prevented with a well-known security measure such as *digital signature* for checking the integrity of the message on the subscriber side.

A more challenging task is to ensure that no more than *k* shares end up at any single broker down the publication delivery path when (*k*, *n*) threshold scheme is enforced. If a broker that received *k* shares happens to be Byzantine, then it can drop all the shares, leaving only *k*-1 shares at the virtual node. In such a case, the original key cannot be reliably reconstructed on the subscriber side. If multiple Byzantine brokers collude each other to collect at least *k* shares, then these Byzantine brokers can reconstruct the secret and abuse it. Therefore, it is important to make sure that Byzantine brokers at every hop do not collectively receive more than *k* shares.

As the very first step, we impose a basic secret share propagation scheme called (*k*, *n*) *threshold propagation*. The implementation of this propagation scheme is provided in Algorithm 1. This algorithm is designed in such a way that no broker on the next-hop virtual node receives more than *k* shares among the *n* split secret shares.

**Algorithm 1:** Deterministic (*k*, *n*) threshold propagation scheme

 /* Input: *n* initial shares generated with (*k*, *n*) secret sharing scheme */

1 **foreach** *Forwarding broker* *B*_*f*_ ∈ *V*_*f*_ **do**

2  *x* = total secret shares *B*_*f*_ has;

3  **foreach** *share* *i* ∈ *x* **do**

4   **foreach** *Receiving broker* *B*_*r*_ ∈ *V*_*r*_ **do**

5    *t* = total received secret shares of *B*_*r*_;

6    **if** *t* < *k* **then**

**Fig 3:**
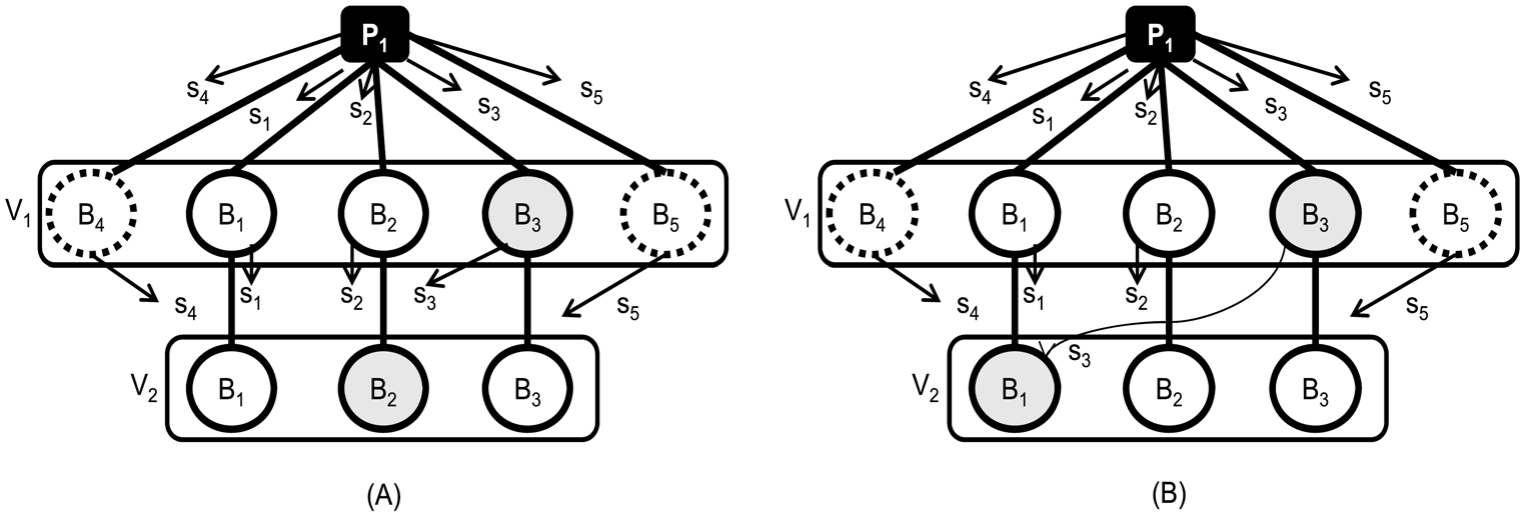
The issues with the propagation of secret shares through multiple hops.

7     send *i* to *B*_*r*_;

8     *t* = *t* + 1;

Algorithm 1 has a serious limitation since a forwarding broker *B*_*f*_ can violate the scheme and arbitrarily send its share to a receiving broker at the next hop, which breaks the requirement that a receiving broker must not receive more than *k* shares out of *n* original split shares. Consider the following example in Fig 3. Publisher *P*_1_ sends out secret shares to brokers *B*_1_, *B*_2_ and *B*_3_ at the virtual node *V*_1_. Now suppose the secret shares have to be relayed to the succeeding virtual node *V*_2_ that also has three replicas. In an ideal case, each broker in *V*_2_ should receive just one share. However, assume that *B*_3_ of *V*_1_ and *B*_2_ of *V*_2_ happen to be Byzantine brokers, as shown in Fig 3(A). *B*_3_ of *V*_1_ may ignore the secret share propagation policy and forward its shares to *B*_2_ of *V*_2_. Upon the receipt of the two shares, *B*_2_ of *V*_2_ may either reconstruct the secret key or intentionally drop the keys. In order to prevent this case, we may consider strengthening the secret sharing scheme at *V*_1_ as follows. The threshold is increased from (2, 3) to (3, 5), and two more replicas (*B*_4_ and *B*_5_) are deployed at *V*_1_ as shown in Fig 3(A). In this way, *B*_2_ at *V*_1_ cannot reconstruct the secret, and other non-Byzantine brokers can safely forward the three shares that are sufficient for reconstructing the original secret. However, we can trivially come up with a case where this new threshold scheme also fails. As shown in Fig 3(B), suppose that *B*_1_ instead of *B*_3_ at *V*_2_ turns out to be the Byzantine broker. Also, suppose that another Byzantine broker *B*_3_ of *V*_1_ forwards its share to *B*_1_ of *V*_2_. These examples show that it is not possible to prevent the situation where *k* shares arrive at an arbitrary single broker at any hop. There are two reasons for this. First, the brokers at the forwarding node cannot detect with certainty whether a replica in the next hop is Byzantine. Second, Byzantine brokers can yield an arbitrary behavior such as sending shares to any replicas on the next hop.

**Fig 4:**
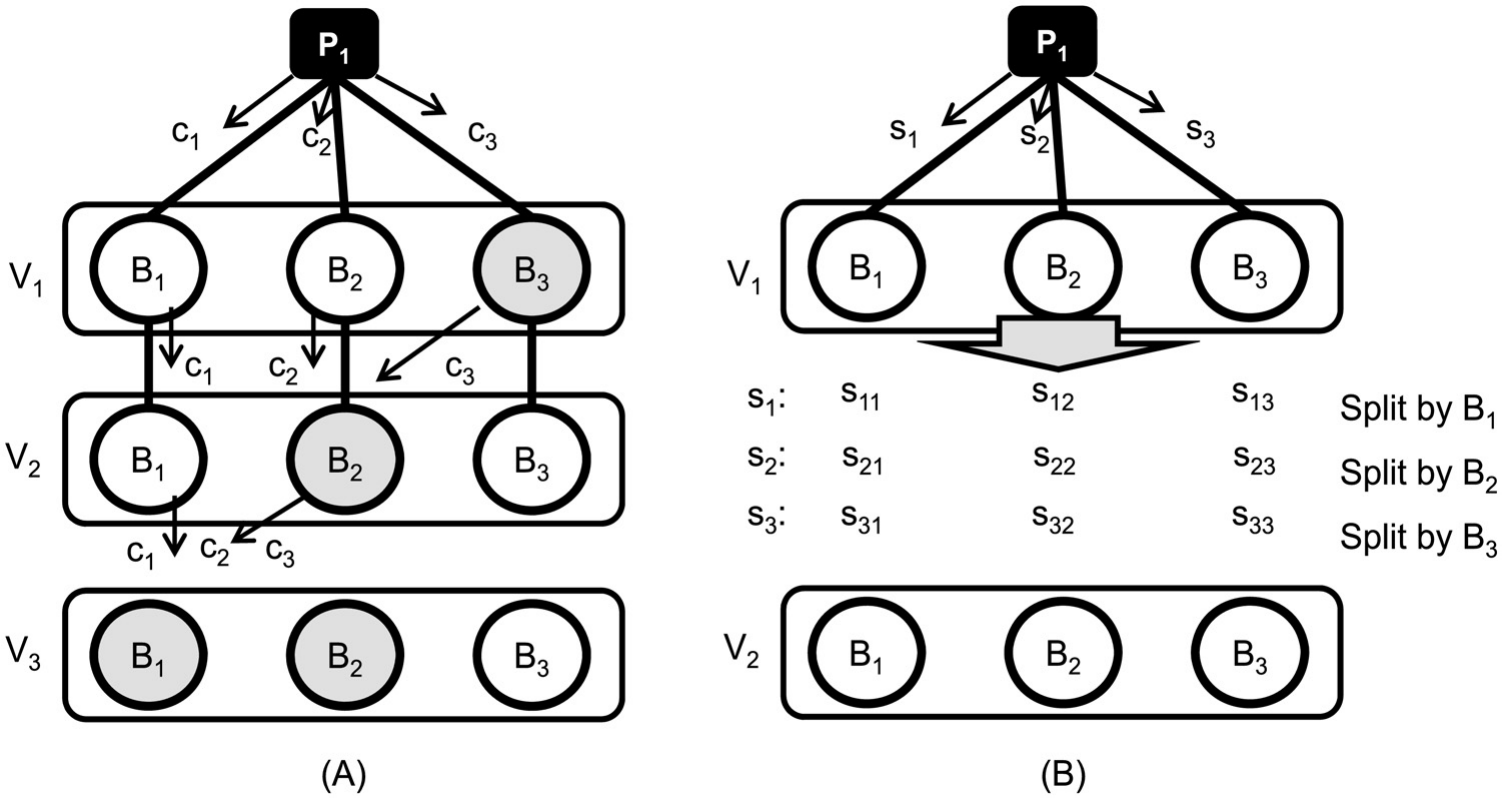
An example of failed delivery of encoded publication messages and an example of guaranteed reliable propagation of secret shares.

As an alternative, we considered splitting the original secret using linear network coding [25]. With linear network coding, a secret is split into *n* encoded blocks. We distribute the *n* encoded blocks according to the propagation scheme in Algorithm 1. This is seemingly a stronger mechanism for preventing the Byzantine brokers to illegally reconstruct the original secret, as the Byzantine brokers need the *entire* *n* encoded blocks to decode the original secret. If (*k*, *n*) threshold scheme is used on the other hand, then only *k*+1 shares are needed for the Byzantine brokers to reconstruct the original secret. If we assume that the majority of the forwarding brokers on a virtual node is non-Byzantine, then it is impossible for a Byzantine broker to receive all *n* encoded blocks as long as the non-Byzantine brokers abide by the propagation scheme described in Algorithm 1. The linear network coding paired with Algorithm 1 may exhibit a higher fault-tolerance. However, this version of secret propagation can also fail. For example, as shown in Fig 4(A), suppose a decryption key is encoded into three blocks, *c*_1_, *c*_2_ and *c*_3_. Assume that *c*_2_ and *c*_3_ reach *B*_2_ of *V*_2_ as the Byzantine broker *B*_3_ of *V*_1_ arbitrarily sends his blocks of code to *B*_2_ instead of *B*_3_ of *V*_2_. There is no concern that *B*_2_ of *V*_2_ will be able to reconstruct the original decryption key. However, this broker may arbitrarily forward all the encoded blocks to *B*_1_ of *V*_3_ at the next hop, which leads to a situation where all the necessary encoded blocks are collected. If *B*_1_ of *V*_3_ turns out to be Byzantine, then this broker can reconstruct the key and use it for any malicious intent.

Note that the aforementioned propagation scheme above splits the original secret only *once* at the publisher. We now opt for splitting a secret share further down the path, and we prove that this is the most viable solution. For example, as shown in Fig 4(B), after *B*_1_ of *V*_1_ receives a secret share *s*_1_ from publisher *P*_1_, it further splits the share into three sub-shares as *s*_11_, *s*_12_ and *s*_13_. Then, *B*_1_ of *V*_1_ relays those further split secret shares to the next hop *V*_2_.

This propagation scheme called *iterative secret propagation* is implemented in Algorithm 2.

**Algorithm 2:** Iterative secret share propagation scheme

 /* Input: *n* initial shares generated with (*k*, *n*) secret sharing scheme */

1 **foreach** *Forwarding broker* *B*_*f*_ ∈ *V*_*f*_ **do**

2  *x* = total secret shares *B*_*f*_ has;

3  **foreach** *share* *i* ∈ *x* **do**

4   *S*(*i*) = List of secret shares by splitting *i* with (|*V*_*r*_|—|*Byz*(*V*_*r*_)|,|*V*_*r*_|) threshold scheme;

5   *x* = first index of *V*_*r*_;

6   **foreach** *i*′ ∈ *S*(*i*) **do**

7    send *i*′ to *x*’th broker in *V*_*r*_;

8    *x* = *x*+1;

We prove that under Algorithm 2 Byzantine brokers in a virtual node cannot receive a sufficient number of secret shares to reconstruct the original secret. Before we proceed with the proof, we set a few additional key assumptions.

**Assumption 2** *Publishers behave correctly*.

In contrary to Assumption 2, publishers can attack publish/subscribe overlay. For example, Wun *et al.* presented the possibility of publishers participating in the denial-of-service attack [26]. However, note that this paper is focused on handling the issues with Byzantine brokers, and devising the security measures against the malicious publishers is not in the scope of this paper.

**Assumption 3** *Non-Byzantine brokers abide by the message propagation rules*.

Now we prove that Theorem 1 holds if Algorithm 2 is enforced by the brokers,

**Theorem 1** *A broker B in V*_*r*_ *cannot receive more than* $\left\lfloor \frac{|V_{f}|}{2}\right\rfloor$ *sets of secret shares from V*_*f*_ *such that, for every set* S *B received, reconstruct*(S) *holds*.

**Proof 1** *Suppose B in V*_*r*_ *received* $\lfloor \frac{|V_{f}|}{2}\rfloor +1$ *sets of secret shares from V*_*f*_, *such that, for every set* S *B received, reconstruct*(S) *holds. This occurs only if* $\lfloor \frac{|V_{f}|}{2}\rfloor +1$ *Byzantine brokers in V*_*f*_ *violate the protocol in Algorithm 2 that a broker must distribute its split shares* S′ *in such a way that reconstruct*(S′) *does not hold (as enforced in Algorithm 2: 8–10). This implies that the majority of the brokers in V*_*f*_ *are not non-Byzantine. Therefore, it contradicts Assumption 1*.

Theorem 1 states that Byzantine brokers cannot reconstruct a secret unless they receive *all* split secret shares, which is not possible given our assumptions.

**Theorem 2** *Non-Byzantine brokers in V*_*r*_ *receive secret shares from the non-Byzantine brokers in V*_*f*_ *that precedes V*_*r*_ *which are sufficient for reconstructing original secret* S *generated by a publisher PUB*.

We prove Theorem 2 by mathematical induction as follows.

**Proof 2** ***Basis***: *There is only one virtual node between PUB and the interested subscribers*.

*Assume that the number of secret shares the non-Byzantine brokers in V*_*r*_ *receive is less than* $\lfloor \frac{|V_{r}|}{2}\rfloor +1$. *This implies that the publisher did not generate a sufficient number of secret shares. Hence, this contradicts Assumption 2*.

***Inductive Step***: *Assume that non-Byzantine brokers in i’th consecutive virtual nodes from PUB receive m secret shares in total that are sufficient for reconstructing the original secret* S. *We show that in the subsequent virtual node V*_*i*+1_, *non-Byzantine brokers receive a sufficient number of secret shares to reconstruct* S.

*Assume that the total number of secret shares the nByz*(*V*_*i*+1_) *received is less than* ($\lfloor \frac{\left|m\right|}{2}\rfloor +1$)($\lfloor \frac{|V_{i+1}|}{2}\rfloor +1$) *from the Byz*(*V*_*i*_). *Note that every non-Byzantine broker nByz in V*_*i*_ *must generate a total of* |*V*_*i*+1_| *secret shares for every secret nByz received from the B*(*V*_*i* − 1_). *We assumed that a correct number of secret shares are received by the non-Byzantine brokers up to i’th virtual node as the inductive step. Therefore, the assumption that the non-Byzantine brokers in V*_*i*+1_ *received less than* ($\lfloor \frac{\left|m\right|}{2}\rfloor +1$)($\lfloor \frac{|V_{i+1}|}{2}\rfloor +1$) *implies that at least one non-Byzantine broker in V*_*i*_ *generated less than* $\lfloor \frac{|V_{i+1}|}{2}\rfloor +1$ *for one of the shares it received from B*(*V*_*i* − 1_). *This also means that the non-Byzantine broker violated the rule specified in Algorithm 2, and therefore it contradicts Assumption 3*.

Theorem 2 states that non-Byzantine brokers are guaranteed to always forward a set of secret shares that are sufficient for reconstructing the original secret at the authorized receiver. Finally, we can derive Theorem 3.

**Theorem 3** *A non-authorized subscriber* (*SUB*2) *that is not entitled to the messages published by a publisher PUB cannot receive a sufficient number of secret shares from the brokers to reconstruct the original key* S *generated by PUB. An authorized subscriber* (*SUB*1) *that is entitled to the messages published by PUB must receive a sufficient number of secret shares to re-construct the original key generated by PUB*.

**Proof 3** ***Basis***: *There is only one virtual node V between PUB and the two subscribers, SUB*1 and *SUB*2.

*Assume that the number of secret shares SUB*1 *receives is insufficient to re-construct* S. *This assumption implies that non-Byzantine brokers in V failed to send sufficient number of secret shares. Therefore, this assumption contradicts Theorem 2*.

*Assume that the number of secret shares SUB*2 *receives is sufficient to re-construct* S. *This assumption implies that Byzantine brokers in V were able to collude each other to send a sufficient number of secret shares to SUB*2 *for re-constructing* S. *However, the number of secret shares PUB sent to Byz*(*V*) *is less than* $\lfloor \frac{\left|V\right|}{2}\rfloor +1$. *Therefore this assumption contradicts Assumption 2 and Assumption 1*.

***Inductive step***: *Assume that Theorem 3 holds when there are i consecutive virtual nodes between PUB and the two subscribers, SUB*1 *and SUB*2. *Show that Theorem 3 holds when there are i* + 1 *virtual nodes between PUB and the two subscribers, SUB*1 and *SUB*2.

*Assume that the number of secret shares SUB*1 *receives via V*_*i*+1_ *is insufficient to reconstruct* S. *This assumption implies that non-Byzantine brokers in V*_*i*+1_ *failed to forward a sufficient number of secret shares to SUB*1. *This contradicts with Theorem 2*.

*Assume that the number of secret shares SUB*2 *receives is sufficient to reconstruct* S. *This can occur only when the* $\lfloor \frac{|V_{i+2}|}{2}\rfloor +1$ *brokers in V*_*i*+1_ *sent their shares to SUB*2. *This indicates that the majority of the brokers in V*_*i*+1_ *is Byzantine, which contradicts Assumption 1*.

In order to reconstruct the original key, a subscriber should know how many virtual nodes the secret shares traversed and how many replicas are allocated at each virtual node. The number of virtual nodes corresponds to the number of reconstructions to apply on the received secret shares. The number of replicas at every virtual node gives a subscriber the necessary information about what threshold scheme to apply when executing the reconstruction. Publishers and brokers tag these pieces of information to the secret shares while the brokers forward them down the end-to-end path towards the subscribers.

#### Solution Analysis and Adaptations

Assume that a decryption key is sent along with every publication. Then, the maximum number of split secret shares a subscriber receives at the end will be at most *pr*^*h*^ where *p* is the number of disjoint end-to-end paths from the publishers to the subscribers, *r* is the average number of replicas at each hop on the end-to-end path and *h* is the path length measured as a hop count. Suppose *f* is the average number of Byzantine brokers at each hop. Then the minimum number of secret shares a subscriber receive at the end is *pr*^*h*^ − *p*(*r* − *f*)^*h*^. The number of secret shares can increase significantly as the path length increases. However, with the rise of Cloud-based pub/sub systems [27], pub/sub overlays are getting flatter, i.e., the end-to-end path length is at most 3.

However, if the number of secret shares is a non-negligible concern, there are two adaptation techniques to reduce the secret shares. One is to refresh the decryption key for a bulk of publications instead of generating one for every publication.

Another adaptation technique is to deliver the secret shares out of band through an external repository. This repository can also be replicated to hold the secret shares separately. This may incur less traffic increase compared to the in-band delivery of secret shares. However, because subscribers have to pull the keys from the repository through another communication channel, opening up the publication content can be delayed further. In contrast, the in-band secret delivery method incurs no additional latency, since the publication content can be opened up immediately with the decryption key that is piggybacked on the publication. The in-band delivery approach also adheres to the nature of pub/sub that the clients are decoupled in time and space to ensure scalable communication [1].

### Propagation of Encrypted Content

So far, we have introduced a decryption key propagation method that is applied to a pub/sub overlay with replicated brokers. Note that replicas introduce multiple alternative routes through which the encrypted content can be forwarded. Typically alternative routes offer opportunities for traffic load-balancing. However, in our context, those routes entail a new issue with the reliable delivery of publication content itself. Given the next hop virtual node with *n* replicas, a forwarding virtual node can prepare *n* duplicates of the encrypted content in order to guarantee the reliable delivery. Replicating the content in such a way down the path towards the interested subscribers can significantly increase network traffic. To avoid this problem, we can opt for sending only one publication to one of the replicas and re-transmit the publication in case it gets lost. However, re-transmitting the publication may incur non-negligible delay. In order to reduce the redundant traffic and delay caused by the re-transmission, we can encode and split the file into multiple blocks and then send them out at the same time through multiple paths. Only the missing blocks need to be re-transmitted. There can be a situation where all the *n* blocks end up in the hands of Byzantine brokers at a certain virtual node. This is still safe because the blocks are encrypted and can only be decrypted with the keys that are transferred securely through the secret share propagation method that we devised in the previous section. For the case of the Byzantine brokers corrupting the blocks, publishers and subscribers can use a *digital signature* mechanism to check the integrity of each block. Overhead of this approach is measured in the evaluation section of this paper.

## The Management of Broker Replicas

In the previous section, we took advantage of the replicas across the broker overlay in order to secure the publication propagation. In this section, we present a novel framework and protocols for managing these replicas.

### Replica Placement Framework

In practice, fully replicating every node in a pub/sub overlay may not be feasible due to cost and limited budget. Therefore, we devise a framework that directs the placements of replicas strategically on the most appropriate locations in a pub/sub overlay for the efficient usage of resources. In our framework, we allow administrators to explicitly specify the criteria for the replica placements. These criteria are mainly broken into two categories. The first criteria specifies the reliability factor (**R**). The second one specifies the performance factor (**P**). With the placement of additional replicas, a pub/sub overlay becomes more fault-tolerant. On the other hand, the addition of the replicas can degrade performance since secret sharing at the replicas increases the latency and traffic, which potentially leads to congestion. To strike the balance between the two contradicting problems above (i.e., reliability versus performance), we have devised a 3-phase allocation method. The input to this method is the set of the end-to-end paths between all publisher and subscriber pairs. Given *n* nodes in an overlay, there can be at most *n*(*n* − 1) end-to-end paths. In the first phase, our framework allocates replicas based on the reliability criteria. A priority is assigned to every end-to-end path. The priority (*ρ*) is measured as a *product* of the following metrics on the end-to-end path: (1) path length measured as the number of hops; (2) failure frequency ratio over a fixed period of time (*γ*) and (3) user-defined weight (*ω*). The failure frequency ratio (*γ*) is the fraction of the number of failures that have occurred on an end-to-end path over the total number of failures occurred on all end-to-end paths. The weight (*ω*) indicates the importance of an end-to-end path, and the user (the administrator) can freely assign a numeric value to it. The replicas are allocated *proportionally* to *ρ*.

**Fig 5:**
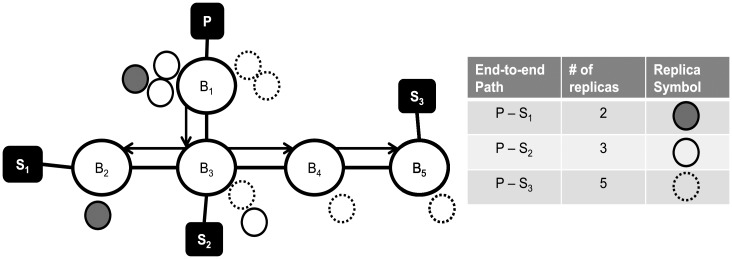
An example of replica placement for the end-to-end path from publisher P to subscribers *S*_1_, *S*_2_ and *S*_3_. *B*s represent brokers. With the replicas, *B*s form a virtual node.

In the second phase, the replicas allotted for each end-to-path are now distributed among the nodes that constitute the end-to-end path. The replicas are distributed proportionally to the failure frequency ratio within the end-to-end path. This frequency ratio of a node on the end-to-end path is measured as the fraction of the number of failures by the node over the total number of failures among all the nodes within the end-to-end path. Fig 5 illustrates a sample placement of replicas after the completion of the second phase for the end-to-end paths between publisher *P* and the subscribers *S*_1_, *S*_2_ and *S*_3_. Assume that the *ρ* values for the paths, *P* − *S*_1_, *P* − *S*_2_ and *P* − *S*_3_ are 2, 3 and 5, respectively, are given. Given 10 available replicas in total, the number of replicas for each path is determined in the first phase, as shown in the table in Fig 5. In the second phase, replicas are assigned to the nodes based on their individual failure frequency ratio. We observed a couple of interesting things about this phase. First, there can be cases where a virtual node consists of only two replicas. In such cases, secret sharing cannot be enforced because we cannot assure that a majority of the nodes will be non-Byzantine. Second, all 3 end-to-end paths intersect at *B*_1_ and *B*_2_. Thus, those two brokers receive a batch of replicas more than once during the execution of the second phase. A possible variation of the second phase is to assign a pack of replicas only once to a node. For example, the 2 packs of replicas can be removed from *B*_1_ and be re-assigned to any under-provisioned nodes.

In the third phase, replicas on each end-to-end path are de-allocated based on how the currently allocated replicas degrade the performance. The main QoS metric we focus on is the latency incurred by the secret sharing scheme. The latency can grow proportionally to the number of replicas. Therefore, the administrators can set the maximum number of replicas that can be assigned per disjoint end-to-end path. If the number of replicas assigned to an end-to-end path in the first phase exceeds the maximum number of replicas allowed, then the replicas can be incrementally removed. Here, we remove the replicas from the node with the least failure frequency ratio first. Note that, this is based on a basic performance model that reflects the correlation between the addition of replicas and the latency metric. Our framework can be extended to employ more sophisticated analytical performance models such as queueing network models [28].

### Dynamic Replica Deployment Protocol

In this section, we provide a protocol for flexibly re-deploying brokers across the pub/sub broker overlay *at runtime*. This re-deployment protocol involves attachment and/or detachment of brokers. This protocol is designed in such a way to prevent disruptions to the publication delivery service. Upon the attachment or the detachment of replicas, the threshold scheme for secret sharing gets updated among the broker replicas on the virtual nodes.

**Fig 6:**
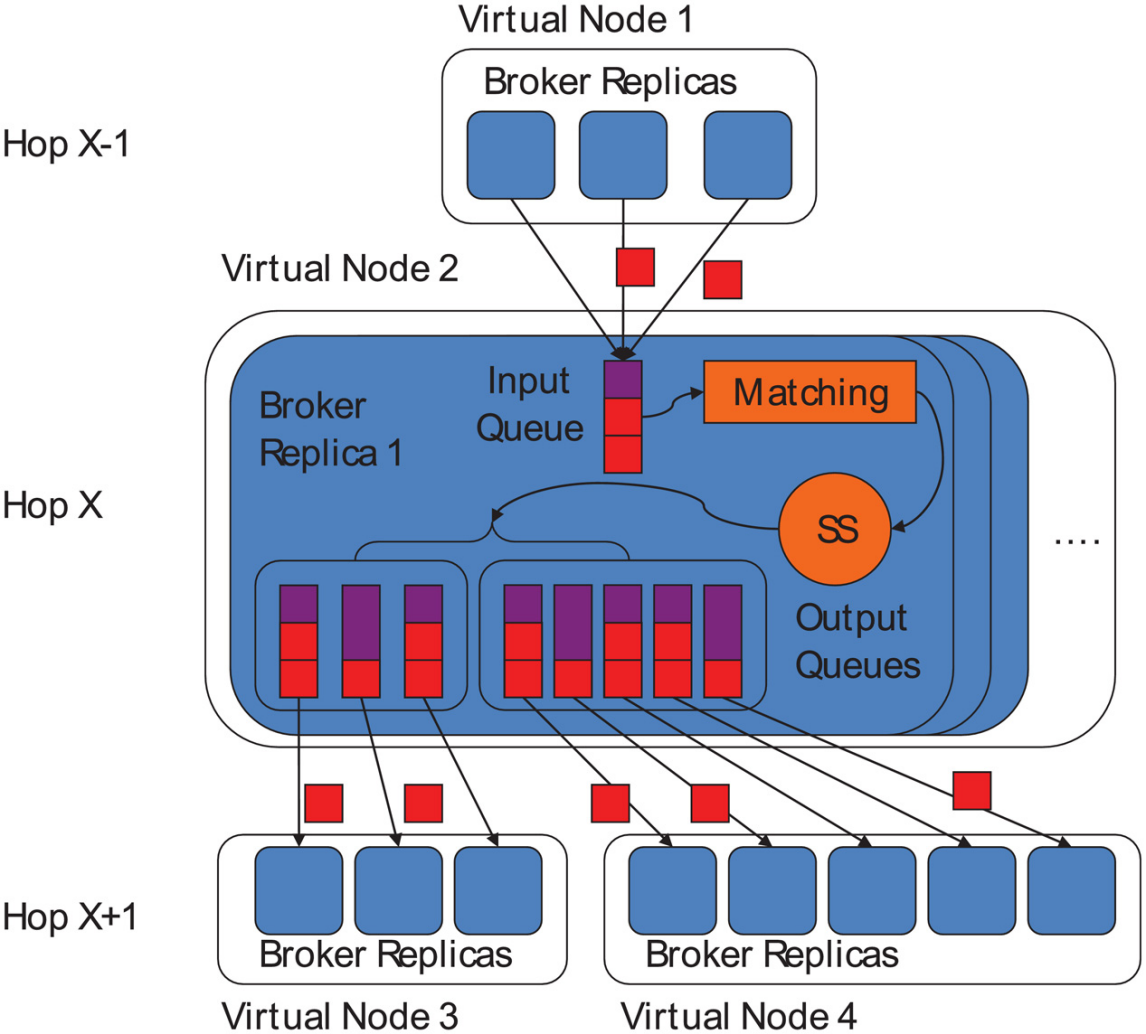
The architecture of the extended pub/sub broker for secret forwarding.

Before we articulate the re-deployment protocol, we describe the extended broker architecture of the reference pub/sub overlay implementation [20, 21]. As shown in Fig 6, each broker has a single input queue and multiple output queues. Output queues are grouped to be associated with each virtual node in the next hop. Each output queue is designated to a broker replica in the next-hop virtual node. A broker receives secret shares from the previous virtual node through its input queue. When a secret share from the previous hop gets dequeued from the input queue, the broker runs a *topic*-based matching in order to determine where the secret shares and publications should be forwarded to. The topic does not need to be encrypted as long as it does not reveal private information. However, if the topic has to be encrypted as well, then homomorphic matching techniques have to be used as introduced in [29], which is the subject for future work. Upon the detection of the next virtual node to forward the secret, a broker first splits the received secret share once again.

**Algorithm 3:** Broker replica detachment

 /* For a detaching broker *B*_*d*_: */

1 Sends its own ID to the all *forwarding* virtual nodes for *B*_*d*_ (N);

2 **while** *Not received ACK from every V* ∈ N **and** *input queue not empty* **do**

3  Keep processing messages in the input queue;

4  Enqueue output messages into appropriate output queues;

5 Flush all output queues;

6 Disconnect from all forwarding and receiving virtual nodes for *B*_*d*_;

**Algorithm 4:** Broker replica attachment

 /* For an attaching broker *B*_*a*_: */

1 Initialize an input queue;

2 Replicate routing state;

3 Configure output group and output queues;

4 Connects with *forwarding virtual nodes* for *B*_*a*_;

5 Notifies the *forwarding virtual nodes* the ID of *B*_*a*_ and a new threshold scheme;

**Algorithm 5:** Updates at a broker in the forwarding virtual node for a broker *B*

 /* When received a notification message */

1 **if** *Received detachment notification* **then**

2  Change threshold scheme;

3  Flush the output queue mapped to B;

4  Remove the output queue mapped to B;

5  Send ACK to the next hop;

6 **else**

 /* When attach notification received:                       */

7  Create and map an output queue to B;

8  Change threshold scheme;

Now we explain the protocol for re-deploying brokers as specified in Algorithm 3, Algorithm 4 and Algorithm 5. Here we provide a couple of definitions that specify a relationship between virtual nodes. A virtual node *V*_*x*_ is a *forwarding virtual node* for a virtual node *V*_*y*_ if *V*_*y*_ sends publications to *V*_*x*_. On the other hand, a virtual node *V*_*x*_ is a *receiving virtual node* for a virtual node *V*_*y*_ if *V*_*x*_ receives publications from *V*_*x*_. Acknowledgements exchanged between the brokers are denoted as ACK.

For the detachment of a broker *B*_*d*_, the brokers in the *forwarding virtual nodes* for *B*_*d*_ have to update the threshold scheme for secret sharing. If *B*_*d*_ is detached, the number of brokers in the virtual node *B*_*d*_ belongs to (denoted as *V*_*B*_*d*__) gets decremented by 1. Suppose the threshold scheme running at the *forwarding virtual nodes* for *B*_*d*_ was originally ($\lfloor \frac{r}{2}\rfloor +1$, *r*) assuming that the number of broker replicas is *r* at *V*_*B*_*d*__. Upon the detachment of *B*_*d*_, the brokers in the *forwarding virtual nodes* should newly enforce a ($\lfloor \frac{r-1}{2}\rfloor +1$, *r* − 1) threshold scheme. The only disruption is caused when the brokers in the *forwarding virtual nodes* cease to process incoming messages when the threshold scheme is updated. However, the update process is executed instantly, thus the disruption is negligible. This update task is highly critical to the confidential delivery of secret keys. If the brokers at the *forwarding virtual nodes* do not pause the processing of incoming messages, then an incoming message may be split with the old threshold scheme. This can cause a case where the majority of secret shares can reach a single broker, which may result in the reconstruction of the secret in case the broker is Byzantine. After the threshold scheme is updated, the brokers at the *forwarding virtual nodes* continue to process the incoming messages as well as flush the output queue mapped to the detaching broker replica.

Likewise, there is a very brief pause at the *forwarding virtual nodes* for attaching broker *B*_*a*_. Similarly, the threshold scheme must change from ($\lfloor \frac{r}{2}\rfloor +1$, *r*) to ($\lfloor \frac{r+1}{2}\rfloor +1$, *r*+1). As stated in Algorithm 4:2, the attaching broker *B*_*a*_ has to replicate one of the brokers at the virtual node to which *B*_*a*_ newly belongs (denoted as $V_{B_{a}}$). We do not employ any popular VM cloning tools such as REMUS [30] and Snowflock [31] for promptly replicating the VM where the broker to replicate might reside. This is because VM cloning replicates the VM state including the security-sensitive information such as the secret share. Because of the security hole in the VM cloning techniques, we resort to adapting the on-demand replication technique of constructing the routing state of the newly attached broker [24]. Subscription and advertisement topics are the ingredients for constructing the complete routing state at *B*_*a*_. *B*_*a*_ can ask any broker in the neighboring virtual nodes to forward their subscriptions and advertisements. However, there can be Byzantine brokers at these neighboring virtual nodes. The Byzantine broker may arbitrarily drop or corrupt the advertisements and subscriptions, thus sabotaging the replication effort by *B*_*a*_. Hence, *B*_*a*_ has to accept only the subscriptions and the advertisements that are sent by the non-Byzantine brokers. Note that *B*_*a*_ cannot identify which broker at $V_{B_{a}}$ is Byzantine. However, we assume that the majority of *r* brokers are non-Byzantine. Therefore, *B*_*a*_ accepts a subscription or an advertisement only if it is received at least $\lfloor \frac{r}{2}\rfloor +1$ times where *r* is the number of replicas at $V_{B_{a}}$. This procedure of checking the validity of subscription and advertisement topics may contribute to the delay in replicating the complete routing state for *B*_*a*_. However, we employ the technique that allows the publications to be delivered at the neighboring brokers of *B*_*a*_ promptly after matching subscriptions are added at *B*_*a*_. Thus, publication delivery resumes very quickly. The non-disruptive nature of our dynamic replica deployment protocol is based on the technique developed in [24]. It allows the broker placements to be revised dynamically if necessary, as explained in the previous section.

## Performance Evaluation

In this section, the performance of our solution is evaluated. We fully integrated the secret sharing in PADRES [20] which is one of the reference implementation of pub/sub overlays. We measured the overhead of our scheme in terms of latency and traffic volume. We empirically assessed the tradeoff between different variations of our scheme, under the presence of Byzantine brokers.

We used Facebook traces that were introduced in [32]. These are the logs of interactions among Facebook users over a 12-month period. There are 3 million anonymous users with 28.3 million relations in total. Note that the data we used in our study was originally collected by the authors of [32], through Facebook’s Graph API. We contacted the authors to obtain the dataset which was collected for research purpose, and the mode of collection fully complies with the Terms and Conditions of Facebook. We assumed that the anonymous Facebook users in this dataset are connected as subscribers to one of the 400 brokers in a pub/sub overlay. Given the placement of the Facebook subscribers, we replayed the interactions in the logs with our new message forwarding scheme enabled.

### The Effect of Secret Sharing on Latency and Traffic

**Fig 7:**
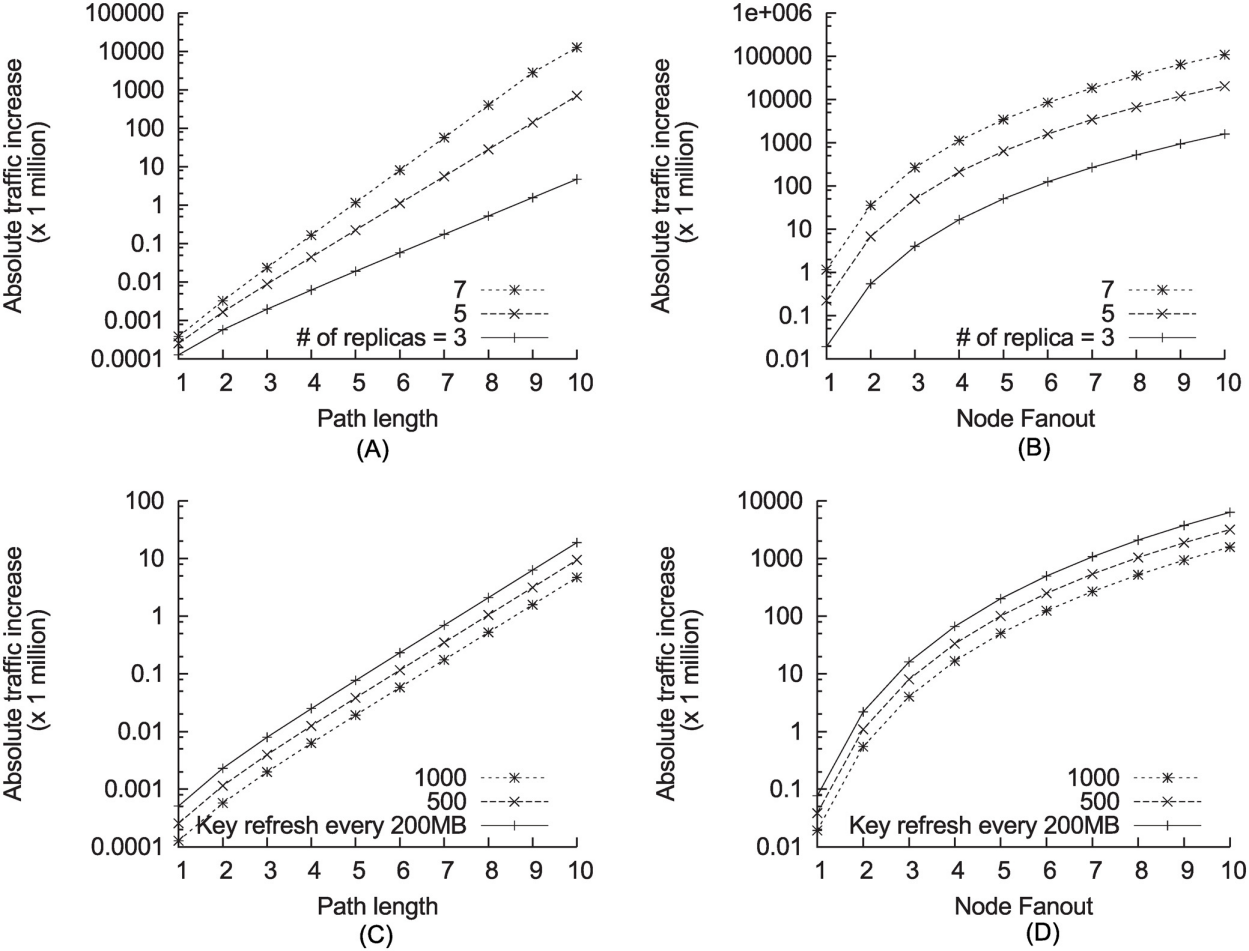
The effect of secret forwarding on latency and traffic with varying node fanout and path length.

Our Java implementation of Shamir’s secret sharing scheme [23] is integrated into the PADRES pub/sub broker. We ran this broker on a machine with Intel Core2 Duo CPU T5550 at 1.83 GHz and 3GB memory. We first measured the number of secret shares as the path length between publishers and subscribers increase. The number of secret shares increases exponentially as the path length and the node fanout increase, as shown in (Fig 7(A) and 7(B)). As mentioned earlier, to reduce the secret shares, publishers can refresh the decryption key for a bulk of publications instead of generating one for every publication. For example, as shown in (Fig 7(C) and 7(D)), the traffic increase can be approximately 10 times less when keys are refreshed every 1GB as oppose to refreshing the keys every 200MB of data.

**Fig 8:**
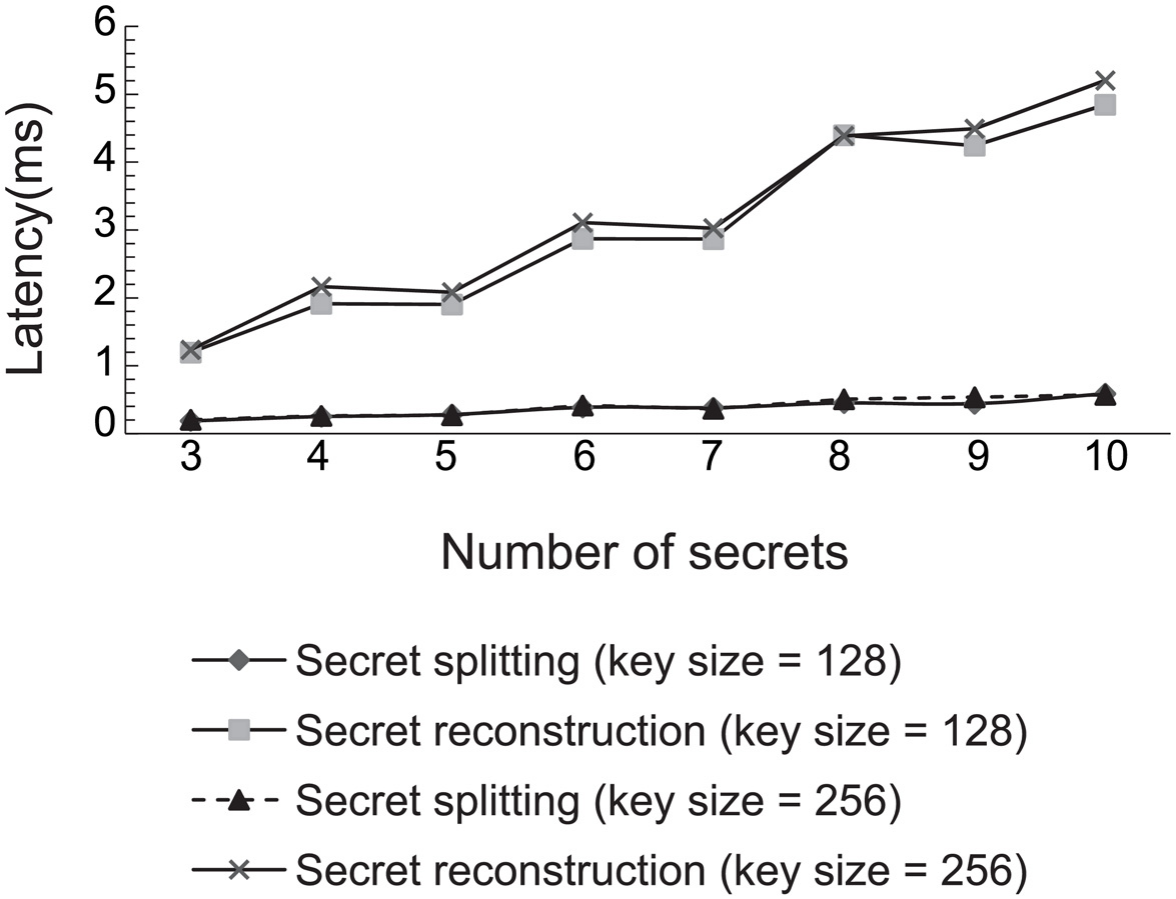
Performance overhead of secret splitting and reconstruction.

We also measured the latency in splitting a secret into 3 to 10 shares. We measured the latency in reconstructing the secret as well. The secret is either an AES-128 or an AES-256 key. We set the threshold scheme (*k*, *n*) where $n=\lfloor \frac{k}{2}\rfloor +1$. As shown in Fig 8, the time it took to split the secret was well under 1 ms. The time it took to reconstruct the secret increased proportionally to the number of shares. It took longer to reconstruct than to split the keys. For example, at the maximum of 10 shares, it took just 5.2 ms on average. However, the reconstruction is done only once by the subscriber, and the brokers along the end-to-end path do not involve in the reconstruction. Our scheme requires the secret splitting at every virtual hop. Thus, the secret splitting overhead is incurred at the broker replicas at every virtual hop. This causes the overall end-to-end latency to increase with the number of hops. However, the end-to-end latency does not grow with the number of replicas at every virtual hop, because the secret splitting is done concurrently among the broker replicas.

### The Effect of Friends Dispersion

**Fig 9:**
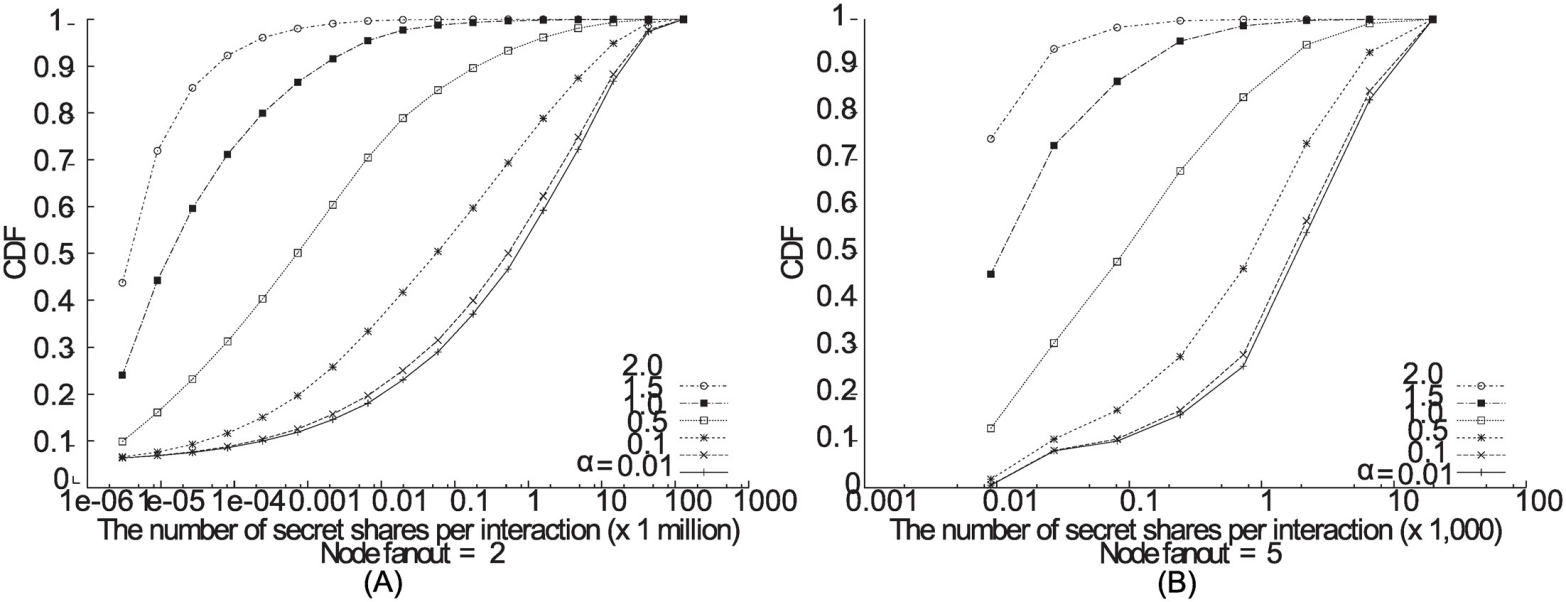
The CDF of secret shares generated per interaction that randomly takes place on a 400-node pub/sub broker overlay.

Facebook users may have friends who are dispersed on many geographical locations. That is, some user may have friends scattered all around the globe, and some user may only have friends from the local region. We conducted a set of experiments on the effect of scattered friends on the overhead of our scheme. We generated a fully connected pub/sub broker overlay topology that does not contain any redundant path. This overlay was assumed to be deployed on a wide area network. Given the overlay, we randomly assigned Facebook users to brokers according to a Zipf distribution. The degree of skewness is controlled by the variable *α*. With a high *α* value, Facebook users are clustered close together. On the other hand, with a low *α* value, Facebook users are disperse and relatively far from each other. We first generated a 400-node overlay with the average node fanout of 2. At every node we assigned 3 replicas that follow the (2, 3) threshold scheme. Fig 9(A) shows the cumulative distribution function of the total secret shares that are delivered during an interaction between a publisher and a subscriber via brokers, over a one-month period. With *α* = 0.5, the median secret shares generated was approximately 80,000. However, the average was 5 million. The pairs of publishers and subscribers that were far apart contributed significantly to this high average. On average, the publishers and the subscribers were 10 hops away from each other with *α* = 0.5. With a higher *α* value of 2, the average number of secret shares per interaction dropped sharply to 860 as the publishers and the subscribers were apart 2.6 hops on average. From this result, we affirmed that the end-to-end path length between the publishers and the subscribers affects the overhead of our scheme. This can guide the administrator of pub/sub broker overlays to reduce the number of hops by consolidating the brokers along the publication delivery paths, so that the number of secret shares is reduced. Also, the node fanout can be controlled to adjust the structure of the overlay. For example, the node fanout of the previous overlay were changed to 5, and the number of brokers are kept the same. Fig 9(B) shows that the number of secret shares per interaction was significantly decreased compared to the case in Fig 9(A). For the same *α* value of 0.5, the case in Fig 9(B) exhibited 939 secret shares generated on average for each interaction. This was a 99% decrease of secret shares compared to the case where the 400-node overlay had a node fanout of 2.

**Fig 10:**
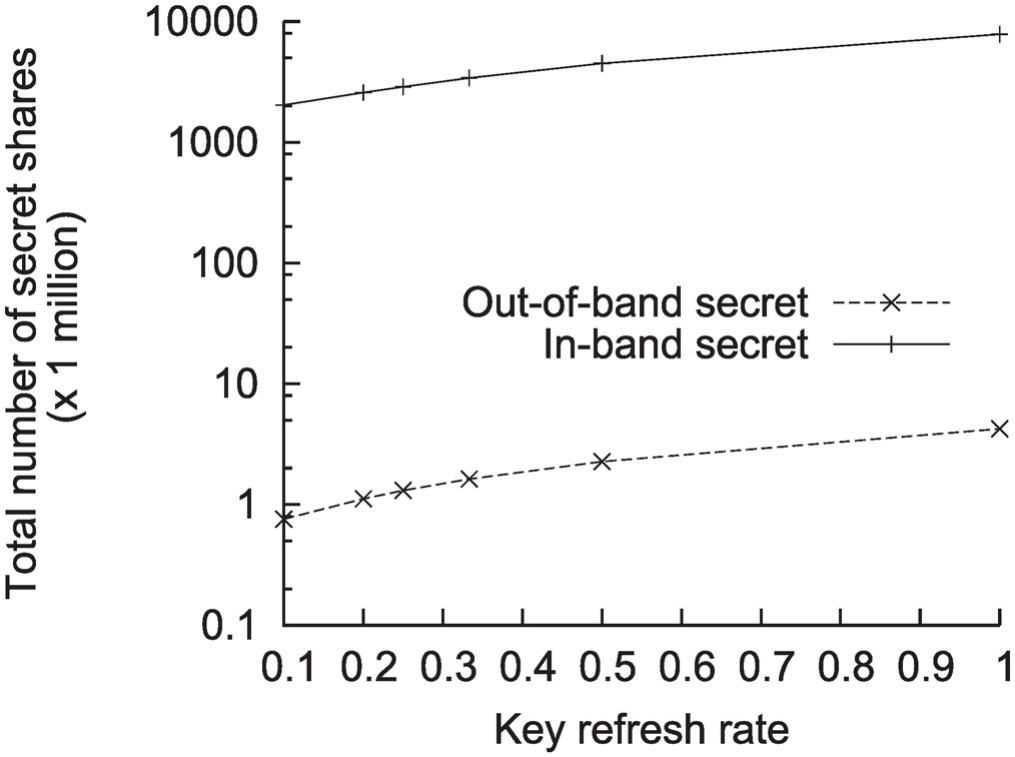
The effect of varying secret key refresh rate.

We also measured the proportion of the secret shares in the data received at the subscriber end. Since we did not know exactly what content was exchanged during the interactions, we could only assume the content was of a certain size. Suppose the average volume of the content per interaction was 1MB. There were a total of 1.4 million interactions over the month in the previous example. Therefore, the total throughput over the month was 1.3 TB/month when our scheme was not applied. With our scheme enabled on a network of 400 nodes with *α* = 0.5 and node fanout of 5, the monthly throughput of secret shares would be approximately 144 GB/month. Hence, the secret share traffic would constitute 11% of the total throughput. This proportion can be a useful indicator of how costly our scheme can be. In order to reduce the proportion of the secret shares, we can refresh the secret key less frequently than refreshing the key for every single message. In Fig 10, the key refresh rate was set to $\frac{1}{m}$ where *m* is the number of messages ranging from 1 to 10. With the key refresh rate of 0.1 and all other settings kept same as the previous example, the monthly secret share traffic throughput was reduced to 39 GB/month. The traffic was reduced by 79% compared to the case where the key refresh rate was 1. With a lower refresh rate, the overlay becomes more vulnerable to the compromise of secret keys. However, the performance overhead could be greatly reduced. This is a simple approach of dealing with the trade-off between the performance and reliability.

Another adaptation technique is to place an *out-of-band* repository for the secret shares used by multiple subscribers. By using this technique, the secret shares could be significantly reduced compared to the scheme of sending the secret shares *in-band*, as shown in Fig 10. However, this adaptation technique has different security and reliability implication as mentioned earlier.

### The effect of Byzantine brokers

**Fig 11:**
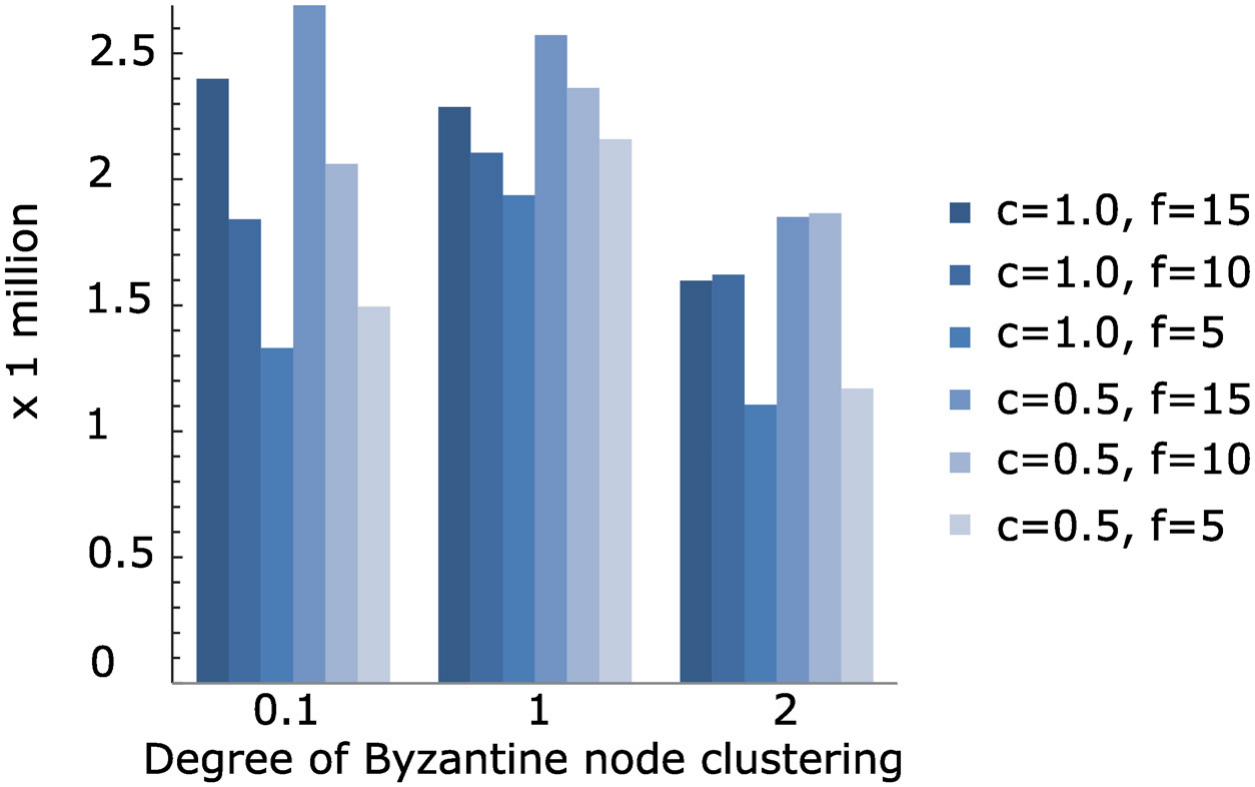
The number of re-transmissions under the presence of Byzantine brokers. *c* indicates how close Facebook users are clustered together. *α* indicates how close Byzantine brokers are clustered together. *f* is the number of randomly chosen Byzantine brokers in the overlay network.

In this section, we show how many re-transmissions can occur under the presence of Byzantine brokers that are randomly chosen from the nodes on the broker overlay network according to the Zipf distribution. We varied the degree of clustering of the Byzantine brokers by varying *α* which determines the skewness of the Zipf distribution. The higher the *α* gets, the closer the Byzantine brokers are clustered together. Similar to the previous test cases, we randomly placed the Facebook users on the pub/sub broker overlays according to the Zipf distribution controlled by the value *c*. We assumed that Byzantine brokers drop messages in order to violate the reliable delivery requirement. We also assume that the failure detection and failover mechanism was not enabled. Given this setting, we replayed the interactions on a 400-node broker overlay with a node fanout of 5, and the result is shown in Fig 11. The first thing we observed is that the increase of re-transmission is not proportional to the increase of the number of Byzantine brokers. This is because a broker may sit on the intersection of multiple end-to-end paths between Facebook users. Therefore, even a small number of Byzantine brokers can affect the pub/sub interactions significantly. When the Facebook users are far apart from each other, there is a higher chance of Byzantine brokers intersecting on different end-to-end paths. However, in a number of cases, re-transmission occurred the most frequently when the Byzantine brokers were moderately dispersed at *α* = 1. This was because the brokers closer to the core of the overlay network were chosen, thus affecting relatively larger numbers of intersections. The total number of re-transmissions was higher than the number of total interactions we replayed. For example, with *α* = 1, *c* = 0.5 and *f* = 15, the number of required re-transmissions exceeded 2.5 million. This indicates that more than one broker along the end-to-end paths between the Facebook users failed during an interaction. From this experiment, we can see that a small fraction of the brokers can affect the pub/sub overlay and the running services significantly. In order to prevent this, a prompt detection of the Byzantine brokers and failover mechanism should be devised. However, *perfectly* detecting a Byzantine broker is not practically feasible. A viable solution is to force the brokers to replicate the received piece of content further down the path as secret share is split further down the path. However, the content can be much larger than the secret shares. Thus, the traffic increase caused by this solution can be impractical. Hence, a new solution that addresses the trade-off between an imperfect failover mechanism and the rigorous replication scheme is needed. We plan to develop this solution in the future.

## Related Work

In this section, we survey how the existing works address the problems with regards to the reliability and confidentiality for pub/sub messaging through the broker overlays. We present novelty of our work by making comparisons to these state-of-the-art works.

Gryphon [33] is a pub/sub system that maintains multiple redundant overlays. If failures occur on an overlay, publishers and subscribers make transition to a backup overlay. Gryphon guarantees exactly-once delivery. However, Gryphon requires over-provisioning of resources for the backup overlays that can be under-utilized most of the time. In contrast, Yoon *et al.* devised a technique to replicate a faulty broker on demand [24]. Upon dynamic replication of a broker, publication delivery can be resumed in various ways to satisfy diverse functional and non-functional requirements. This work also supports exactly-once delivery and per-publisher FIFO ordering. However, this work is mainly focused on replicating a single faulty broker. In [34], Kazemdzadeh *et al.* devised a pub/sub system where each broker has configurable visibility of its neighbors. For example, if the visibility is set to 3, a broker can access the state of the neighbors that are up to 3 hops away. In this system, a broker can adaptively establish a soft link to bypass multiple faulty brokers that are within its scope of visibility. However, the last two aforementioned works [24, 34] do not support confidential delivery of publications. Therefore, they cannot prevent unwanted subscribers from receiving and disclosing the content of the publication. Our pub/sub system executes secret forwarding technique in order to conduct confidential delivery as well as reliable delivery. Similar to the work in [24], our pub/sub system is based on overlay that can elastically grow and shrink as oppose to over-provisioning redundant overlays such as the systems in [33, 34]. Note that our work is more advanced than the pub/sub system in [24], as our pub/sub system can tolerate more than one faulty brokers.

A few existing pub/sub works protect confidentiality of the contents in the publication using access control mechanisms. For example, Gryphon provides the access control scheme for limiting who may publish and subscribe to portions of the information space. EventGuard [35] supports access control as well. EventGuard assumes a threat model where routing brokers can eavesdrop and drop or flood messages while publishers and subscribers are reliable. However, these works do not address the case where multiple Byzantine brokers collude each other to disclose private contents in publications. Our system prevents such case by enforcing secret sharing scheme on the brokers. In [36], role-based access control is used to enforce access control transparently among the brokers and clients. However, this work trusts the brokers to act correctly, whereas we account for the case where a broker can be Byzantine.

Another line of work for protecting publication confidentiality uses cryptographic techniques for encrypting publications. Since the publication is encrypted, even if malicious brokers or unintended subscribers receive the publications, they cannot disclose the confidential content unless they possess a decryption key. However, this method faces a non-trivial dilemma to resolve. Although this method may prevent data leakage to a certain degree, it makes content-based routing challenging as examining the actual content is not possible with encrypted contents. In order to solve this dilemma, Nabeel *et al.* [37] derived a set of attributes from the content of the publications and ran the matching algorithm over these attributes instead of the encrypted payload. Also, Nabeel *et al.* used *homomorphic* encryption techniques to execute the matching operations over the encrypted publication content without the need of decryption. The result of matching using this method is ensured to be consistent with the methods of matching over the non-encrypted publications. In [38], Choi *et al.* focused on reducing the performance overhead of matching homomorphically encrypted publications against subscriptions at the brokers using a *scalar product preserving transformation*. However, this line of work requires the publishers and subscribers to contact each other in advance to exchange decryption keys through out-of-band channels, similar to the work presented in [39]. Unlike our approach, this breaks the unique nature of pub/sub that the clients are normally decoupled in time and space [1]. These works do not address the case where the broker with homomorphic matching capability suffer crash failure. Our pub/sub system is tolerant to crash failures, since our system dynamically adds brokers to the virtual nodes on demand. Our system is orthogonal to these secure content-matching techniques.

In [40], shared secret is proposed to protect the authenticity, integrity and confidentiality of publication from the untrusted brokers and subscribers. However, this work is based on a centralized security infrastructure that manages the shared secrets. This centralized approach can limit the scalability of pub/sub systems. Moreover, the centralized security manager can become a single point of failure. Our work does not assume any central repository for storing shared secrets. In our pub/sub system, secrets are forwarded through the pre-deployed distributed brokers. Therefore, there is no need to introduce additional infrastructure to manage secrets. Our work also protects secrets even in the case where multiple Byzantine brokers reside along the publication propagation paths, through iterative secret propagation technique.

So far, we learned that state-of-the-art works have applied conventional security techniques such as replication, access control and encryption to pub/sub systems. However, to the best of our knowledge, none of the existing works addresses the case where decryption keys can get compromised by the Byzantine brokers, which is a serious threat to the secure delivery of private publications. Also, oftentimes, these existing works rely heavily on expensive synchronization mechanisms and centralized coordinators, while our work exploits distributed brokers. None of the existing works tackle the case where more than one Byzantine brokers reside along the publication propagation paths. We apply the iterative secret propagation technique to delivery secrets securely through the publication delivery paths. While the existing works focus solely on the security issues, our work provides a framework that helps the administrators to devise the best custom policy for striking the balance between security/reliability and performance/efficiency requirements. Most of the existing works assume over-provisioned redundant broker overlays that cannot flexibly grow and shrink. Our work employs the technique of replicating and consolidating brokers on demand based on configurable security and performance requirements.

## Conclusion

On pub/sub broker-based overlays, we applied the secret forwarding method to broker replicas in order to ensure reliable and confidential delivery of encrypted content and decryption keys. Our method is tolerant to the presence of Byzantine brokers along the delivery path of publications as long as more than half of the broker replicas on each virtual node at every end-to-end path are non-Byzantine. Secret keys are split further at every virtual node down the publication delivery path. This method is proven to prevent the situation where Byzantine brokers can collude to reconstruct the secret key for decrypting confidential messages. This method also prevents publication message drops by the Byzantine brokers. We assessed the performance implication of our scheme on a PADRES pub/sub broker overlay and discussed several adaptations to our scheme. In addition to the secret forwarding technique, we addressed the efficient usage of resources by devising a framework to place broker replicas strategically on different parts of the overlay according to reliability and performance requirements that are configurable. We also implemented a non-disruptive protocol for detaching and attaching broker replicas to realize any update to the placements of broker replicas.
